# Spectroscopic Diagnosis of Arsenic Contamination in Agricultural Soils

**DOI:** 10.3390/s17051036

**Published:** 2017-05-04

**Authors:** Tiezhu Shi, Huizeng Liu, Yiyun Chen, Teng Fei, Junjie Wang, Guofeng Wu

**Affiliations:** 1 Key Laboratory for Geo-Environmental Monitoring of Coastal Zone of National Administration of Surveying, Mapping and GeoInformation & Shenzhen Key Laboratory of Spatial Smart Sensing and Services & College of Life Sciences and Oceanography, Shenzhen University, 518060 Shenzhen, China; tiezhushi@whu.edu.cn (T.S.); zhongzheng0512@126.com (H.L.); wjjlight@whu.edu.cn (J.W.); 2 Department of Geography, Hong Kong Baptist University, Kowloon Tong, Kowloon, Hong Kong, China; 3 School of Resource and Environmental Sciences, Wuhan University, 430079 Wuhan, China; chenyy@whu.edu.cn (Y.C.); feiteng@whu.edu.cn (T.F.); 4 Suzhou Institute of Wuhan University, 215000 Suzhou, China

**Keywords:** visible and near-infrared reflectance spectroscopy, heavy metal contamination, spectral pre-processing, feature selection, machine-learning

## Abstract

This study investigated the abilities of pre-processing, feature selection and machine-learning methods for the spectroscopic diagnosis of soil arsenic contamination. The spectral data were pre-processed by using Savitzky-Golay smoothing, first and second derivatives, multiplicative scatter correction, standard normal variate, and mean centering. Principle component analysis (PCA) and the RELIEF algorithm were used to extract spectral features. Machine-learning methods, including random forests (RF), artificial neural network (ANN), radial basis function- and linear function- based support vector machine (RBF- and LF-SVM) were employed for establishing diagnosis models. The model accuracies were evaluated and compared by using overall accuracies (OAs). The statistical significance of the difference between models was evaluated by using McNemar’s test (*Z* value). The results showed that the OAs varied with the different combinations of pre-processing, feature selection, and classification methods. Feature selection methods could improve the modeling efficiencies and diagnosis accuracies, and RELIEF often outperformed PCA. The optimal models established by RF (OA = 86%), ANN (OA = 89%), RBF- (OA = 89%) and LF-SVM (OA = 87%) had no statistical difference in diagnosis accuracies (*Z* < 1.96, *p* < 0.05). These results indicated that it was feasible to diagnose soil arsenic contamination using reflectance spectroscopy. The appropriate combination of multivariate methods was important to improve diagnosis accuracies.

## 1. Introduction

Soil heavy metal contamination demands effective methods for diagnosing suspected contaminated areas and controlling the rehabilitation process. There is increasing interest in using visible and near-infrared reflectance spectroscopy (VNIRS, 350–2500 nm) to measure soil heavy metal contents and to map its spatial distribution [1], since this technique provides a non-destructive, rapid, and cost-effective method for measuring several soil properties from a single scan, and requires minimal sample preparation and hazardous chemicals [2].

The spectroscopic measurement of heavy metals is usually feasible because of their indirect relationships with some spectral feature soil properties, such as organic matter, iron-oxides or clays [1]. Therefore, the spectral information for soil heavy metal estimations is weak, indirect, and non-specific. Moreover, the spectral features of soil properties in visible/near-infrared spectra are largely overlapping, while other factors, such as surface roughness, moisture content, and organic matter of soil, also weaken the spectroscopic measurement of soil properties [3]. Thus, the analysis of visible/near-infrared spectra requires the use of multivariate chemometric techniques to mathematically extract useful information for soil property estimations.

Pre-processing techniques are commonly used to reduce the random noise, baseline drift and multiple scattering effects in the spectra [1]. For instance, Savitzky-Golay (SG) smoothing is adopted to increase the spectral quality by eliminating random noise. Derivative transformation can remove background interferences, resolve overlapping spectra and minimize the baseline drift caused by the differences in grinding and optical setups [4]. Multiplicative scatter correction (MSC) and standard normal variate (SNV) seek to eliminate the multiplicative interferences of scattering and particle size [5]. Moreover, data enhancement algorithms, such as mean centering (MC) and normalization, are able to highlight the diversities of spectral data, reduce redundant information, and simplify calibration models [5]. For soil reflectance spectroscopy, the type and amount of pre-processing required are data-specific; no single or combination of pre-processing techniques will work well with all data sets [6].

Feature selection techniques, such as successive projection algorithm (SPA), uninformative variables elimination (UVE) and genetic algorithm (GA), are often applied to remove uninformative spectral bands and to select optimal spectral variable subsets for establishing regression models [7,8]. SPA is a forward feature selection technique, and it uses a simple projection operation in a vector space to minimize the collinearity problem [9]. UVE detects uninformative spectral variables based on a stability analysis of regression coefficients (b-coefficient) [10]. GA uses a probabilistic, non-local search process to randomly select an initial spectral data-set and to optimize this data set by considering many combinations of spectral variables and their interactions [10]. In soil spectroscopy, GA always results in better performances than SPA and UVE for soil property estimates [7,8].

These feature selection methods are designed to select features to improve the estimation of numerical variables, such as soil property contents, and they are inappropriate to reduce dimensionality and select features for classifying nominal variables, such as heavy metal contamination levels. Principal component analysis (PCA) and the RELIEF algorithm have been widely applied for feature selection in the classification applications, such as image classification and text categorization [11]. However, as far as we know, PCA and RELIEF have rarely been employed to select features for diagnosing soil heavy metal contamination from soil reflectance spectra.

From a large data-set using trained models, data mining techniques automatically or semi-automatically uncover patterns, which are used on a new data-set for prediction [12]. Various data mining techniques, such as principal component regression (PCR) [13], partial least squares regression (PLSR) [14,15,16], artificial neural network (ANN) [4], multivariate adaptive regression splines (MARS) [17] and support vector machine (SVM) [18,19,20] were employed to train models from spectral data for estimating soil properties, including heavy metals. The ‘training model’ process is synonymously described as ‘machine-learning’, which can be defined as the process of discovering the relationships between predictor and response variables using computer-based statistical methods [21]. In soil science, machine-learning techniques have been used to classify soil types, soil depth classes, and soil drainage classes [22]. However, few studies have adopted machine-learning techniques to diagnose soil heavy metal contamination from soil reflectance spectroscopy [23].

Several studies have adopted multivariate chemometric techniques to quantitatively predict heavy metal contents in agricultural soils by using reflectance spectroscopy. For example, Ren et al. [24] used PLSR to establish a quantitative relation between reflectance spectra and As, and Cu contents in agricultural soils; Wu et al. [13] predicted Hg concentration in suburban agricultural soils of the Nanjing region by using PCR and reflectance spectra within the visible-near-infrared region. By reviewing the literature on soil heavy metal predictions, it is found that the prediction accuracies of soil heavy metal contents usually cannot reach a good quantitative level (the recommended R^2^ of 0.81 or above for soil analysis [25]) because of the indirect prediction mechanisms. For practical applications, such as soil heavy metal monitoring, contamination remediation, or digital soil mapping, the diagnosis of soil heavy metal contamination may be sufficient rather than accurate heavy metal content estimations. However, at present, soil reflectance spectroscopy is rarely employed to qualitatively diagnose soil heavy metal contamination. To the best of our knowledge, Bray et al. [23] were the first to employ an ordinal logistic regression technique to diagnose Cd, Cu, Pb and Zn contamination in urban soils from reflectance spectra. Therefore, it is interesting and necessary to extend the knowledge about the diagnosis of soil heavy metal contamination by using soil reflectance spectroscopy.

In China, arsenic content has continuously increased in agricultural soils during the past 30 years, because of some anthropogenic activities, such as chemical fertilizers, arsenic-bearing pesticides, animal manures, mining, smelting, and irrigation with arsenic-contaminated water [26]. Excessive arsenic accumulation in agricultural soils can hinder the crops’ growth and decrease the yield and quality of agricultural products. Moreover, as a potent carcinogen, arsenic might pose a serious health threat to the human body, such as malignant arsenical skin lesions, respiratory disease, gastrointestinal disorder, liver malfunction, nervous system disorder and haematological diseases [27].

Given the importance of monitoring arsenic contamination in agricultural soils, this study aimed to compare the abilities of pre-processing techniques (derivative transformations, MSC, SNV, MC) and machine-learning techniques (random forests (RF), ANN, and SVM) in diagnosing soil arsenic contamination from soil reflectance spectroscopy, and to investigate whether the feature selection approaches (PCA and RELIEF) could improve the diagnosis accuracy by using different machine-learning methods. The result of this study is expected to establish a technical process for diagnosing soil heavy metal contamination by using soil reflectance spectroscopy.

## 2. Materials and Methods

### 2.1. Soil Samples

**Figure 1:**
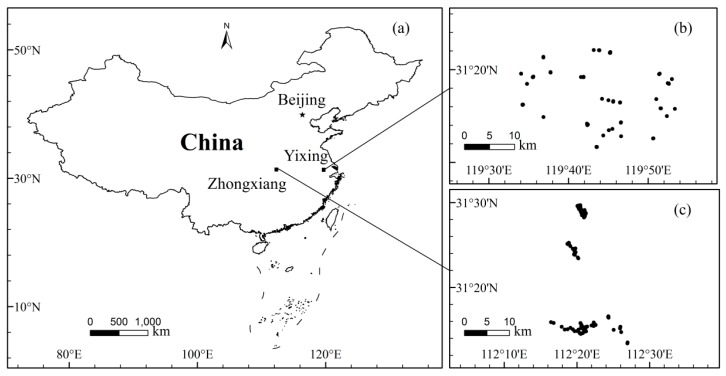
Study areas (**a**) and spatial distribution of soil samples in Yixing (**b**) and Zhongxiang (**c**).

In total, 195 historical soil samples collected in Yixing and Zhongxiang regions were used for this work. Yixing (Figure 1b) is located in the south of Jiangsu Province, China, with an annual temperature of 15.7 °C and a mean annual precipitation of 1177 mm. Zhongxiang (Figure 1c) is situated in the middle of Hubei Province, China, and its mean annual temperature is 15.0 °C with a mean annual precipitation of 961 mm. Yixing’s dominant soil types are dystric cambisols, lixisols, anthrosols, alisols, calcaric fluvisols, calcisols, cambisols and gleysols for different crop cultivation [20]. The soils collected from Zhongxiang mainly belong to anthrosols for rice planting [28]. At each sample site, surface soils (0–10 cm) were collected. The industrial wastewater, exhaust gas or waste residues produced by local chemical factories are the major causes of arsenics contamination in agricultural soils in the Zhongxiang region [28]; in Yixing, the contamination may mostly result from sewage irrigation, parent materials or vehicle exhausts [29].

### 2.2. Laboratory Spectrum and Soil Arsenic Content Measurement

Soil samples were air-dried and ground in a mechanical agate grinder to a particle size of ≤2 mm. The diffuse reflectance spectra were measured by using the FieldSpec3 portable spectroradiometer (ASD Inc., now PANalytical Company, Boulder, CO, USA) with a spectral range of 350 to 2500 nm. The spectral measurements were conducted in a dark room. The air-dried and ground soil sample was placed in a 10 cm diameter petri dish with a thickness of approximately 15 mm. A 50 W halogen lamp was used as the light source, which was positioned 30 cm away from soil sample, with a 15° zenith angle [20]. The optical probe was installed about 15 cm above the soil sample. A Spectralon panel (Labsphere, North Sutton, NH, USA) was used for white referencing once every six measurements.

After spectral measurement, soil samples were further ground, and passed through a 100-mesh sieve (0.15 mm). The finely ground soil samples were digested by HF-HClO4-HNO3. The arsenic contents of digested samples were then analyzed by using a hydride generation atomic fluorescence spectrometry (HG-AFS) method [30]. Certified soil reference materials (GBW 07401, GBW 07402, and GBW 07407, National Research Center for Certified Reference Materials of China) were used to verify the precision of HG-AFS method.

For the purpose of diagnosis, the measured soil arsenic contents were coded into binary 0 or 1, describing uncontaminated or contaminated samples, respectively. The index of geo-accumulation (I_geo_) [31] was applied to assess the arsenic contamination in the soils:

(1) $$I_{\mathrm{geo}}=\log_{2}\frac{M_{\mathrm{As}}}{1.5B_{\mathrm{As}}}$$

where M_As_ is the measured arsenic contents in the soils, B_As_ is the geochemical background value of arsenic (13 mg·kg^−1^), the constant of 1.5 was used to eliminate fluctuations caused by regional differences and anthropogenic influences [31]. I_geo_ ≤ 0 indicates practically uncontaminated, whereas I_geo_ > 0 means contaminated [31].

### 2.3. Pre-Processing Transformations

The whole measured soil arsenic content data and their corresponding spectral data were divided into training (*n* = 98) and test (*n* = 97) data sets using a Kennard-Stone algorithm [32], which is effective for selecting spectra-representative samples for model development. The reflectance spectra were first reduced to 400–2450 nm to remove the wavelengths with high noise effects at the spectral edges. The reflectance spectra were then SG smoothed with a moving window of 9 nm. The smoothed spectra were resampled to 10 nm intervals (e.g., 400, 410, and 420 nm, etc.) to eliminate the data redundancy by using a Gaussian model [4]. Moreover, first and second derivatives, MSC, SNV and MC of reflectance spectra were performed for soil spectra to enhance spectral features and to further establish robust diagnosis models. Reflectance spectra were transformed into log(1/Reflectance) before MSC and SNV were performed.

### 2.4. Feature Selection

PCA and the RELIEF algorithm were applied to extract features from spectral variables of the training data-set. PCA was an optimal linear scheme for extracting several principle components (PCs) from high dimensional variables, and the extracted components can hold the majority of the variables’ information. The RELIEF algorithm, first described by Kira and Rendell [33], was used as a simple, fast and effective approach to weigh variables, and its output is the ranking weights between −1 and 1 for spectral variables, in which the more positive weights indicate more predictive spectral variables. In this study, PCA and the RELIEF algorithm were implemented in Weka (Waikato Environment for Knowledge Analysis). The number of PCs was determined by the diagnosis accuracy of the calibration. The threshold for the RELIEF weight value was set to 0, and the scattered spectral bands with local extreme weights were selected as spectral features to avoid the multicollinearity among RELIEF-selected features.

### 2.5. Multivariate Diagnosis Analysis

Machine-learning methods, such as RF, ANN and SVM, were employed for calibrating diagnosis models using the training data set. For brevity, the summaries of these techniques were provided, and some key references were cited. Interested readers may find more details about these techniques in these references. In this study, the machine-learning methods were implemented by using a R-based Rattle package developed by Williams [34].

#### 2.5.1. Random Forests (RF)

RF, introduced by Breiman [35], is an ensemble learning method that constructs a multitude of decision trees. For the RF learner, each tree is independently trained from a randomized bootstrap sample of the entire training data set, and a subset of explanatory variables is randomly selected for the node-splitting rules in each tree [36]. In classification, trees are voted by majority [35]. The RF depends only on two user-defined parameters: the number of variables in each random subset (*nv*) and the number of trees in the forest (*nt*). In this study, the *nv* was optimized from 1 to the total number of variables with increments of 1, and *nt* from 0 to 1000 by increments of 10. The variable that is important for RF modeling can be determined by mean decrease GINI values.

#### 2.5.2. Artificial Neural Network (ANN)

The concept of ANN learner may date back to 1940s when McCulloch and Pitts [37] initially planned to develop a virtual “central nervous system” for computer modeling. The design of ANN simulates the data processing in biological nervous systems. The structure of an ANN consists of a set of interconnected neurons. Some neurons are adopted for the reception of information, others for its forwarding and storage, and another group for the outward release of information [38]. Neurons are connected to each other through weighted synapses. In an ANN, the number of hidden layers and neurons in each hidden layer ought to be optimized [21]. In this study, the number of hidden layers was optimized by iterating this parameter from 1 to 20, and the number of neurons in each layer was set as the total number of variables.

#### 2.5.3. Support Vector Machine (SVM)

SVM is a kernel-based machine learning method developed on the basis of statistical learning theory [39]. SVM applies a kernel function to map training data into a higher dimensional feature space, and computes separating hyperplanes that achieve maximum separation (margin) between the classes [40]. The maximum separation hyperplane is the training data on the margin, which are called support vectors. The quality of the SVM classifier is affected by the type of kernel function, kernel width (*γ*) and regularization parameter (*C*) [40]. In this study, radial basis function (RBF) and linear function (LF) were adopted as kernel functions, respectively.

### 2.6. Validation and Comparison of Diagnosis Models

The calibrated models were applied for diagnosing the contaminated and uncontaminated soil samples of the test data-set. The overall accuracy (OA, Equation (2)) [38] of the test data-set was calculated and employed for comparing the diagnosis abilities of multivariate methods. The same computer environment was kept for running different machine-learning algorithms.

(2) $$OA=\frac{pp+nn}{pp+np+pn+nn}$$

where the meanings of *pp*, *np*, *pn* and *nn* are displayed in Table 1.

The statistical significance of the difference between diagnosis models was evaluated by using McNemar’s test [41], which is based on a binary distinction between correct and incorrect class allocations (Table 2). McNemar’s test is also based on the standardized normal test statistic expressed in Equation (3):

(3) $$Z=\frac{f_{12}-f_{21}}{\sqrt{f_{12}+f_{21}}}$$

Therefore, the test is focused on the cases that are correctly diagnosed by one classifier but misdiagnosed by the other. Two diagnosis models may exhibit different accuracies at the 95% level of confidence if *Z* > |1.96|.

## 3. Results

### 3.1. Soil Arsenic and the Spectra

The percent mean standard error of the HG-AFS method for arsenic determination was 2.9%. The descriptive statistics of soil arsenic of the 195 soil samples are shown in Table 3. For the total data set, the soil arsenic contents varied from 1.91 to 133.36 mg·kg^−1^, with a mean of 18.13 mg·kg^−1^ and a standard deviation of 18.67 mg·kg^−1^. Considering I_geo_ values, 27%, 26% and 29% of samples were contaminated by arsenic in total, training and test data sets, respectively.

**Figure 2:**
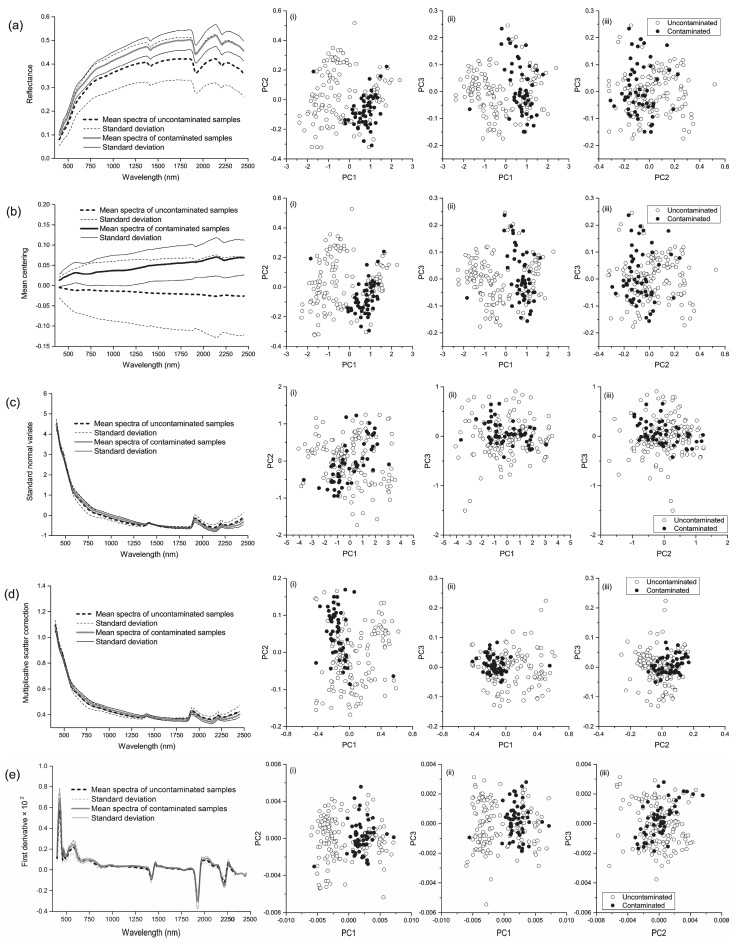

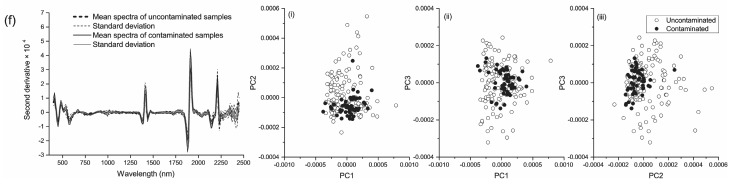
The reflectance spectra and the three first principal components (PC1, PC2 and PC3) for the contaminated and uncontaminated soil samples: (**a**) original reflectance spectra, (**b**) mean centering spectra, (**c**) standard normal variate spectra, (**d**) multiplicative scatter correction spectra, (**e**) first derivative spectra, and (**f**) second derivative spectra.

The mean value and standard deviation of original and pre-processed spectra for contaminated and uncontaminated soil samples are shown in Figure 2. Three prominent absorption peaks around 1400, 1900 and 2000 nm are visibly water absorption features [42] (Figure 2a); MC centered the reflectance spectra on 0 values (Figure 2b); SNV (Figure 2c) and MSC (Figure 2d) had similar spectral curves, and served the same purpose to remove the multiple scattering effects in the reflectance spectra; first (Figure 2e) and second (Figure 2f) derivatives minimized the baseline drift and highlight the minor absorption features of reflectance spectra. These demonstrated that the original reflectance and pre-processed spectra of uncontaminated and contaminated soil samples were overlapped, which indicates that there might exist a nonlinear relationship between spectra and soil arsenic contamination.

### 3.2. Principal Components and RELIEF Selected Features

The first three loadings of the PCA analysis for original reflectance and pre-processed spectra were displayed in Figure 2. The score plots showed that the spectral space of the contaminated samples fell into those of uncontaminated samples. This meant that the linear classifier might be unable to effectively diagnose contaminated or uncontaminated soil samples by using principal components.

**Figure 3:**
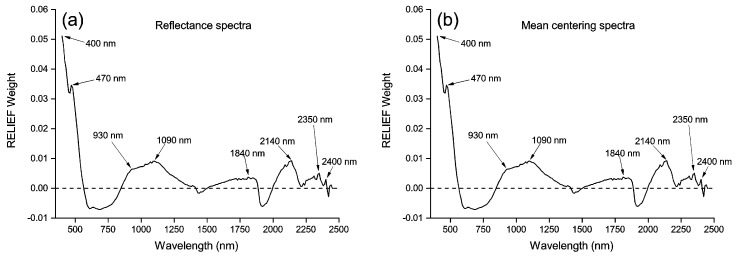

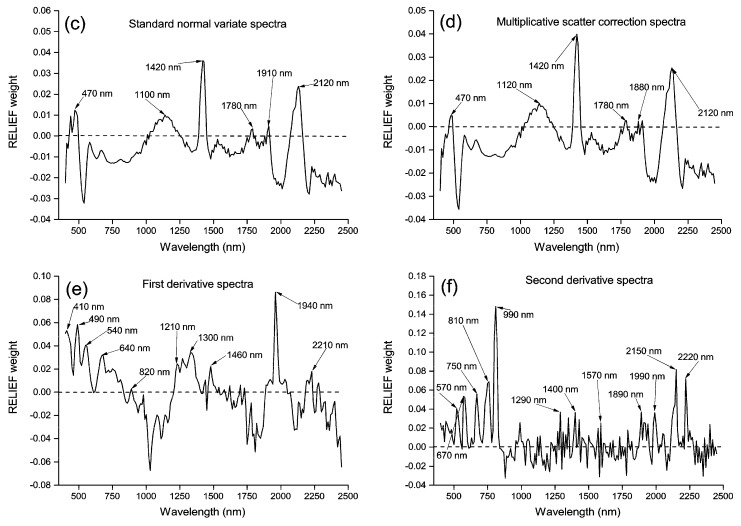
RELIEF weights and the selected spectral features for original reflectance spectra (**a**), mean centering spectra (**b**), standard normal variate spectra (**c**), multiplicative scatter correction spectra (**d**), first derivative spectra (**e**), and second derivative spectra (**f**). The threshold of RELIEF weight was set to 0 (horizontal dashed lines).

The RELIEF weights and the selected spectral features are displayed in Figure 3. The RELIEF weights of the MC spectra (Figure 3b) had the same values as those of original reflectance spectra (Figure 3a), thus the same spectral variables at 400, 470, 930, 1090, 1840, 2140, 2350 and 2400 nm were selected as spectral features for original reflectance and MC spectra. The RELIEF weights of SNV (Figure 3c) and MSC (Figure 3d) processed spectra showed the same tendency, and the same spectral variables at 470, 1100, 1420, 1780, 1910 and 2120 nm were identified as spectral features. Spectral variables at 410, 490, 540, 640, 820, 1210, 1300, 1460, 1940 and 2210 nm (Figure 3e), and variables at 570, 670, 750, 810, 990, 1290, 1400, 1570, 1890, 1990, 2150 and 2220 nm (Figure 3f) were selected as spectral features for first and second derivatives, respectively. Compared with the original reflectance, MC, SNV and MSC spectra, first and second derivatives resulted in more spectral features with higher RELIEF weights.

### 3.3. Comparison of the Abilities of Different Methods

The operation times, parameter setting, and validated OAs for diagnosis models by using different methods are illustrated in Table 4. The results showed that (1) the suitable combination of pre-processing and feature selection was vital to improve OAs of each machine-learning method; (2) feature selection methods, PCA and RELIEF, could improve modeling accuracies and decrease operation times of modeling, and RELIEF often outperformed PCA; (3) derivative transformation often resulted in the best diagnosis models. The optimal models for RF, ANN, LF and RBF-SVM were described as follows:

#### 3.3.1. RF

**Figure 4:**
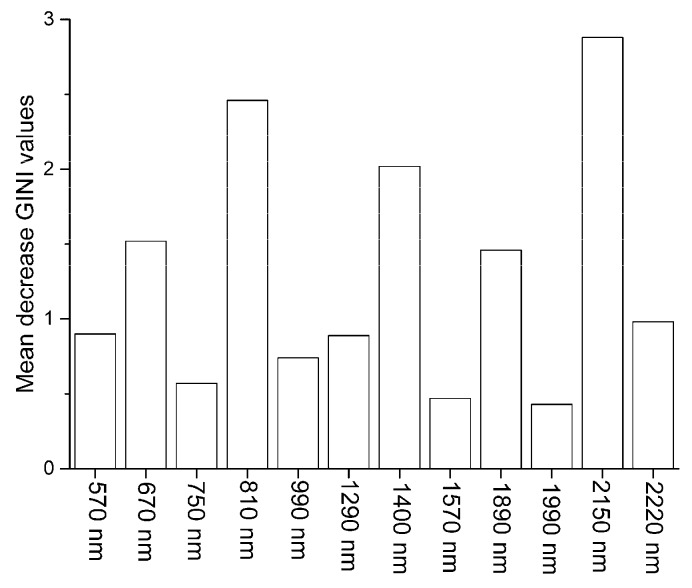
Mean decrease GINI values for RELIEF-selected spectral features.

**Figure 5:**
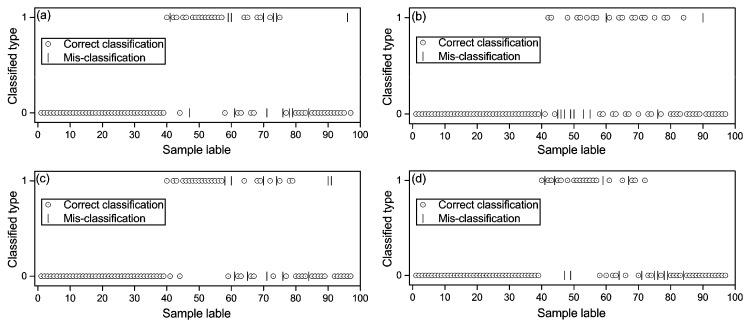
Values of samples predicted by using: (**a**) second derivative spectra (second), RELIEF and random forests; (**b**) first derivative spectra (first), principle component analysis and artificial neural network; (**c**) second, RELIEF and radial basis function-based support vector machine (SVM); and (**d**) first, RELIEF and linear function-based SVM. Value 1 indicates contaminated, and value 0 indicates uncontaminated. The correctly-diagnosed and misdiagnosed samples are displayed in the figures.

The optimal pre-processing method for the RF model was second dervative. The best RF model was calibrated by using 12 RELIEF-selected spectral features, and the optimized *nv* and *nt* of the RF model were 3 and 50, respectively. The mean decrease GINI values (Figure 4) showed the importance of the spectral features for RF modeling in descending order as 2150, 810, 1400, 670, 1890, 2220, 1290, 570, 990, 750, 1570 and1990 nm. The validated OA for the RF model was 86%, which mean that the RF model correctly diagnosed 86% of soil samples in the test data-set (Figure 5a).

#### 3.3.2. ANN

The optimal pre-processing method employed for ANN modeling was first derivative; PCA was selected as the feature selection method, and the number of hidden layers was three. The factor number for modeling was eight, and the first eight PCs explained approximately 99% of the variation of the spectral data. The ANN model correctly diagnosed 89% of soil samples in the test data-set (Figure 5b).

#### 3.3.3. SVM

Second derivative was the optimal pre-processing method for RBF-SVM, and first derivative was the optimal pre-processing method for LF-SVM. The optimized C and γ for RBF-SVM were 1 and 0.06, respectively, while the optimized C for LF-SVM was 1. By adopting 12 RELIEF-selected spectral features, the RBF-SVM model correctly diagnosed 89% of soil samples in the test data-set (Figure 5c); and the LF-SVM model correctly diagnosed 87% of soil samples by using the RELIEF-selected spectral features (Figure 5d).

#### 3.3.4. Model Comparison

Figure 5 displayed the predicted values of samples in the test dat-set by using three optimal diagnosis models. McNemar’s test applied to these diagnosis models showed that the *Z* values were all less than 1.96 (Table 5), which indicated that there was no statistical difference in the diagnosis abilities of these optimal diagnosis models (*p* < 0.05).

## 4. Discussion

In this study, with the combination of pre-processing, feature selection and machine-learning methods, the OAs for soil arsenic contamination diagnosis achieved a satisfactory level (OA > 85%). This result demonstrated that VNIRS could be applied to diagnose soil arsenic contamination, although in the process of developing diagnosis models, VNIRS technology depended on conventional methods for providing the ground-truth of soil heavy metal contamination. Compared with conventional methods, this study confirmed that VNIRS might allow for faster and cheaper classification of soil heavy metal contaminants in an increased spatial coverage, which has been suggested by Bray, Viscarra Rossel and McBratney [23].

This study demonstrated that, to establish robust diagnosis models, the trial and error of various pre-processing methods was vital. Pre-processing methods, including SNV, MSC, first derivative, and second derivative, can be employed to eliminate the baseline drift caused by the difference in particle size and optical setups [6]. Derivative transformations also enhance the minor absorption features which may be useful to improve the diagnosis abilities of models. Nevertheless, derivative transformation will add noises into the spectral data, generating more noises with the increase of derivative orders [20]. Therefore, derivative transformations are often applied in conjunction with a smoothing algorithm to amplify noise [6]. Our research suggested that, compared with other pre-processing methods, derivative transformation was a more suitable pre-processing method for developing diagnosis models.

Feature selection methods could improve modeling accuracies by eliminating uninformative spectral variables and increase modeling efficiency by reducing the independent variables for modeling [10]. PCA extracted principle components from spectral variables without consideration of dependent variables (i.e., soil arsenic contamination in this study). However, RELIEF-selected spectral features based on their contributions to the classification of dependent variables [33]. Therefore, the results in this study indicated that RELIEF always outperformed PCA for diagnosing soil arsenic contamination from hyperspectral spectra. We considered that, based on these factors, the RELIEF algorithm was a more suitable method to select spectral features. Moreover, van Groenigen et al. [43] demonstrated that pre-processing methods could strongly influence the reflectance spectra, and they will therefore have an impact on the spectral features. Therefore, in this study, the results indicated that pre-processing methods affected the RELIEF-selected spectral features (Figure 3).

The establishment of robust diagnosis models by using different machine-learning methods (i.e., RF, ANN, LF-SVM, RBF-SVM) depends on the selection of appropriate pre-processing and feature selection methods. In addition, our study demonstrated that these optimal models for machine-learning methods had no statistical difference in diagnosis abilities; moreover, RF was superior to other machine-learning methods because of its ability to simplify parameter optimization and its better models explanatory. In this study, based on mean decrease GINI values, wavelengths at 2150, 810, 1400, and 670 nm can be identified as the first four important wavelengths for diagnosing arsenic contamination with the RF model. Wavelengths near 2150 and 810 nm relate to organic matter, and spectral features near 1400 and 670 nm coincide with wavelengths related to mostly iron oxides [42]. This might demonstrate that the diagnosis of arsenic contamination might depend on its surrogated correlations with organic matter and iron oxides.

Over-fitting is a common problem for modeling. It means that the best diagnosis model for the training data-set will not work well for the test data-set. RF is robust against over-fitting. Breiman [35] observed that the error associated with the error of RF converged to a limit with the increase in the number of trees in a forest. Nevertheless, in the case of ANN, over-fitting is a serious problem [40]. RF is easily accessible to non-specialists because of its simplicity in parameters optimization. However, for SVM, a number of hyper-parameters need to be optimized for each kernel function [40], while its parameters optimization also requires considerable knowledge of the frequently non-trivial underlying mathematics [40]. Moreover, complex machine-learning algorithms, such as SVM and ANN, were not easily interpretable to present relationships between independent and dependent variables [44]. However, RF, a method that performs a majority vote of tree-based classifiers, is explicit and comprehensible, revealing the important spectral variables for modeling [40]. Variable importance in RF can be evaluated by the increase in prediction error when the out-of-bag data are permuted for a certain variable, while keeping all other data constant. Considering these advantages, we regarded RF as a more efficient machine-learning method for modeling soil arsenic contamination levels.

This study investigated the abilities of laboratory reflectance spectroscopy to diagnose soil arsenic contamination. The field and air-/space-borne imaging spectroscopy have the potential to rapidly map heavy metal contamination over large areas [17,45,46]. Compared with laboratory spectroscopy, the application of field or imaging spectroscopy faces some constraints, such as soil surface, atmospheric and illumination conditions [47]. Therefore, the principles of this study should be further tested with field and imaging data.

## 5. Conclusions

The spectroscopic diagnosis of soil arsenic contamination is feasible, and the appropriate combination of pre-processing, feature selection and machine-learning methods is important for diagnosis accuracies. The RELIEF algorithm is a simple and efficient method to extract spectral features to improve modeling efficiency and diagnosis accuracy. Compared with ANN and SVM, RF is a more optimal machine-learning method for developing diagnosis models, because of its ability to simplify parameter optimization and its better models explanatory.

## Figures and Tables

**Table 1:** Confusion matrix of observed and diagnosed soil samples for calculating overall accuracy ^1^.

Allocation		Observed	
		Contaminated (Positive, Value = 1)	Uncontaminated (Negative, Value = 0)
Predicted	Contaminated (positive, value = 1)	*pp*	*np*
	Uncontaminated (negative, value = 0)	*pn*	*nn*

^1^ *pp*: number of correctly diagnosed contaminated soil samples; *np*: number of falsely diagnosed uncontaminated soil samples; *pn*: number of falsely diagnosed contaminated soil samples; *nn*: number of correctly diagnosed uncontaminated soil samples.

**Table 2:** Assessment of the statistical significance of the difference between two diagnosis models using McNemar’s Test ^1^.

Allocation		Diagnosis Model 2	
		Correct	Incorrect
Diagnosis model 1	Correct	*f*_11_	*f*_12_
	Incorrect	*f*_21_	*f*_22_

^1^ *f*_12_: the test soil samples that are correctly diagnosed by diagnosis model 1 but misdiagnosed by diagnosis model 2; *f*_21_ the test soil samples that are correctly diagnosed by diagnosis model 2 but misdiagnosed by diagnosis model 1.

**Table 3:** Statistical descriptions for the arsenic contents (mg·kg^−1^) and the percent value of contaminated samples (per %) ^1^.

	No.	Minimum	Maximum	Mean	Std.	Per %
Total data set	195	1.91	133.36	18.13	18.67	27
Training data set	98	1.91	106.10	12.70	16.81	26
Test data set	97	4.40	133.36	19.00	20.43	29

^1^ No.: number of samples; Std.: standard deviation.

**Table 4:** The operation times, parameter setting, and overall accuracies for diagnosis models by using different pre-processing, feature selection and machine-learning methods ^1^.

Machine-Learning Methods	Pre-Processing Methods	Feature Selection Methods																						
		No Feature Selection							PCA								RELIEF							
		Parameters					time (s)	OA (%)	*n*_PC_	Parameters					time (s)	OA (%)	*n*_feature_	Parameters					time (s)	OA (%)
		*nt*			*nv*					*nt*			*nv*					*nt*			*nv*			
RF	none	70			5		0.32	80	5	60			7		0.22	82	8	150			5		0.04	85
	MC	270			4		0.27	74	7	160			2		0.17	83	8	130			3		0.03	71
	SNV	290			3		0.32	84	7	20			2		0.05	70	6	60			3		0.03	82
	MSC	150			4		0.25	71	6	30			2		0.03	71	6	30			2		0.03	71
	1st	50			2		0.25	77	8	80			4		0.05	79	10	30			3		0.03	81
	2nd	200			2		0.28	85	6	50			4		0.05	71	**12**	**50**			**2**		**0.05**	**86**
		Parameters					time (s)	OA (%)	*n*_PC_	Parameters					time (s)	OA (%)	*n*_feature_	Parameters					time (s)	OA (%)
		*n*_layer_								*n*layer								*n*layer						
ANN	none	1					0.34	86	6	9					0.05	71	8	3					0.02	84
	MC	2					0.48	76	8	2					0.04	71	8	10					0.05	76
	SNV	1					0.27	81	6	2					0.03	64	6	6					0.03	86
	MSC	1					0.28	29	8	2					0.03	40	6	3					0.02	52
	1st	3					0.67	87	**8**	**3**					**0.03**	**89**	10	1					0.03	81
	2nd	1					0.30	82	5	2					0.03	62	12	1					0.03	75
		Parameters					time (s)	OA (%)	*n*_PC_	Parameters					time (s)	OA (%)	*n*_feature_	Parameters					time (s)	OA (%)
		*γ*	*C*			*n*_sv_				*γ*	*C*			*n*_sv_				*γ*	*C*			*n*_sv_		
RBF-SVM	none	0.01	1			32	0.11	80	7	0.04	1			32	0.05	85	8	0.17	1			32	0.02	82
	MC	0.01	1			32	0.14	70	7	0.08	1			35	0.05	87	8	0.38	1			31	0.03	76
Machine-learning methods	Pre-processing methods	Feature selection methods																						
		No feature selection							PCA								RELIEF							
		Parameters					time (s)	OA (%)	*n*_PC_	Parameters					time (s)	OA (%)	*n*_feature_	Parameters					time (s)	OA (%)
		*γ*	*C*			*n*_sv_				*γ*	*C*			*n*_sv_				*γ*	*C*			*n*_sv_		
RBF-SVM	SNV	0.01	1			36	0.09	81	9	0.04	1			42	0.04	66	6	0.28	1			36	0.03	80
	MSC	0.01	1			37	0.08	71	5	0.23	1			38	0.03	71	6	0.31	1			37	0.02	71
	1st	0.01	1			46	0.06	79	8	0.05	1			43	0.05	75	10	0.09	1			33	0.33	82
	2nd	0.01	1			53	0.08	81	5	0.07	1			41	0.03	71	**12**	**0.06**	**1**			**42**	**0.05**	**89**
		Parameters					time (s)	OA (%)	*n*_PC_	Parameters					time (s)	OA (%)	*n*_feature_	Parameters					time (s)	OA (%)
		*C*		*n*_sv_						*C*		*n*_sv_						*C*		*n*_sv_				
LF-SVM	none	1		36			0.16	84	7	1		35			0.05	81	8	1		37			0.05	80
	MC	1		36			0.12	85	7	1		35			0.05	85	8	1		35			0.03	79
	SNV	1		33			0.11	86	5	1		27			0.06	56	6	1		39			0.06	72
	MSC	1		34			0.11	29	5	1		39			0.06	29	6	1		39			0.04	73
	1st	1		26			0.09	80	8	1		27			0.05	80	**10**	**1**		**29**			**0.05**	**87**
	2nd	1		36			0.10	76	4	1		30			0.06	63	12	1		26			0.05	81

^1^ RF: random forests; ANN: artificial neural network; SVM: support vector machine; RBF: radial basis function; LF: linear function; MC: mean centering; SNV: standard normal variate; MSC: multiplicative scatter correction; 1st: first derivative; 2nd: second derivative; PCA: principle component analysis; time: operation time for calibration; OA: validated overall accuracy; *n*_PC_: number of principle components; *n*_feature_: number of RELIEF selected features. *nt*: number of trees; *nv*: number of variables; *n*_layer_: number of layers; *n*_sv_: number of support vectors. *C*: regularization parameter; *γ*: kernel width. The results of selected models are emphasized in bold.

**Table 5:** *Z* Values of McNemar’s test between the optimal diagnosis models.^1^

	Second + RELIEF + RF	First + PCA + ANN	Second + RELIEF + RBF-SVM
First + PCA + ANN	0.24		
Second + RELIEF + RBF-SVM	0.90	0.00	
First + RELIEF + LF-SVM	0.30	0.26	0.41

^1^ Second: second derivative spectra; First: first derivative spectra; PCA: principle component analysis; RF: random forests; ANN: artificial neural network; SVM: support vector machine; RBF: radial basis function; LF: linear-function.

## References

[1] Shi T.Z., Chen Y.Y., Liu Y.L., Wu G.F. (2014). Visible and near-infrared reflectance spectroscopy—An alternative for monitoring soil contamination by heavy metal. J. Hazard. Mater..

[2] Viscarra Rossel R.A., Walvoort D.J.J., McBratney A.B., Janik L.J., Skjemstad J.O. (2006). Visible, near infrared, mid infrared or combined diffuse reflectance spectroscopy for simultaneous assessment of various soil properties. Geoderma.

[3] Ben-Dor E., Irons J.R., Epema G.F. (1999). Remote Sensing of the Earth Sciences: Manual of Remote Sensing.

[4] Kemper T., Sommer S. (2002). Estimate of heavy metal contamination in soils after a mining accident using reflectance spectroscopy. Environ. Sci. Technol..

[5] Chu X.L., Yuan H.F., Lu W.Z. (2004). Progress and application of spectral data pretreatment and wavelength selection methods in nir analytical technique. Prog. Chem..

[6] Stenberg B., Viscarra Rossel R.A., Mouazen A.M., Wetterlind J., Agronomy A.I. (2010). Visible and Near Infrared Spectroscopy in Soil Science.

[7] Vohland M., Emmerling C. (2011). Determination of total soil organic c and hot water-extractable c from vis-nir soil reflectance with partial least squares regression and spectral feature selection techniques. Eur. J. Soil Sci..

[8] Shi T.Z., Chen Y.Y., Liu H.Z., Wang J.J., Wu G.F. (2014). Soil organic carbon content estimation with laboratory-based visible-near-infrared reflectance spectroscopy: Feature selection. Appl. Spectrosc..

[9] Araújo M.C.U., Saldanha T.C.B., Galvão R.K.H., Yoneyama T., Chame H.C., Visani V. (2001). The successive projections algorithm for variable selection in spectroscopic multicomponent analysis. Chemom. Intell. Lab. Syst..

[10] Zou X.B., Zhao J.W., Malcolm J.W., Holmes M., Mao H.P. (2010). Variables selection methods in near-infrared spectroscopy. Anal. Chim. Acta.

[11] Guyon I., Elisseeff A. (2003). An introduction to variable and feature selection. J. Mach. Learn. Res..

[12] Witten I.H., Frank E. (2005). Data Mining: Practical Machine Learning Tools and Techniques.

[13] Wu Y.Z., Chen J., Ji J.F., Tian Q.J., Wu X.M. (2005). Feasibility of reflectance spectroscopy for the assessment of soil mercury contamination. Environ. Sci. Technol..

[14] Kooistra L., Wehrens R., Leuven R.S.E.W., Buydens L.M.C. (2001). Possibilities of visible-near-infrared spectroscopy for the assessment of soil contamination in river floodplains. Anal. Chim. Acta.

[15] Wu Y.Z., Chen J., Wu X.M., Tian Q.J., Ji J.F., Qin Z.H. (2005). Possibilities of reflectance spectroscopy for the assessment of contaminant elements in suburban soils. Appl. Geochem..

[16] Chen T., Chang Q.R., Clevers J.G.P.W., Kooistra L. (2015). Rapid identification of soil cadmium pollution risk at regional scale based on visible and near-infrared spectroscopy. Environ. Pollut..

[17] Wu Y.Z., Zhang X., Liao Q.L., Ji J.F. (2011). Can contaminant elements in soils be assessed by remote sensing technology: A case study with simulated data. Soil Sci..

[18] Tan K., Ye Y.Y., Du P.J., Zhang Q.Q. (2014). Estimation of heavy metal concentrations in reclaimed mining soils using reflectance spectroscopy. Spectrosc. Spectr. Anal..

[19] Lucà F., Conforti M., Castrignanò A.M., Matteucci G., Buttafuoco G. (2017). Effect of calibration set size on prediction at local scale of soil carbon by vis-nir spectroscopy. Geoderma.

[20] Shi T.Z., Cui L.J., Wang J.J., Fei T., Chen Y.Y., Wu G.F. (2013). Comparison of multivariate methods for estimating soil total nitrogen with visible/near-infrared spectroscopy. Plant Soil.

[21] Heung B., Ho H.C., Zhang J., Knudby A., Bulmer C.E., Schmidt M.G. (2016). An overview and comparison of machine-learning techniques for classification purposes in digital soil mapping. Geoderma.

[22] Brungard C.W., Boettinger J.L., Duniway M.C., Wills S.A., Edwards T.C. (2015). Machine learning for predicting soil classes in three semi-arid landscapes. Geoderma.

[23] Bray J.G.P., Viscarra Rossel R.A., McBratney A.B. (2009). Diagnostic screening of urban soil contaminations using diffuse reflectance spectroscopy. Aust. J. Soil Sci..

[24] Ren H.Y., Zhuang D.F., Singh A.N., Pan J.J., Qiu D.S., Shi R.H. (2009). Estimation of as and cu contamination in agricultural soils around a mining area by reflectance spectroscopy: A case study. Pedosphere.

[25] Vohland M., Besold J., Hill J., Frund H.C. (2011). Comparing different multivariate calibration methods for the determination of soil organic carbon pools with visible to near infrared spectroscopy. Geoderma.

[26] Huang R.Q., Gao S.F., Wang W.L., Staunton S., Wang G. (2006). Soil arsenic availability and the transfer of soil arsenic to crops in suburban areas in fujian province, southeast China. Sci. Total Environ..

[27] Santra S.C., Samal A.C., Bhattacharya P., Banerjee S., Biswas A., Majumdar J. (2013). Arsenic in foodchain and community health risk: A study in gangetic west bengal. Procedia Environ. Sci..

[28] Shi T.Z., Liu H.Z., Wang J.J., Chen Y.Y., Fei T., Wu G.F. (2014). Monitoring arsenic contamination in agricultural soils with reflectance spectroscopy of rice plants. Environ. Sci. Technol..

[29] Wang J.J., Cui L.J., Gao W.X., Shi T.Z., Chen Y.Y., Gao Y. (2014). Prediction of low heavy metal concentrations in agricultural soils using visible and near-infrared reflectance spectroscopy. Geoderma.

[30] Guo J.H., Ma H., Wang S.F. (2009). Determination of arsenic in national standard reference soil and stream sediment samples by atomic fluorescence spectrometry. Rock Miner. Anal..

[31] Loska K., Wiechula D., Korus I. (2004). Metal contamination of farming soils affected by industry. Environ. Int..

[32] Kennard R.W., Stone L.A. (1969). Computer aided design of experiments. Technometrics.

[33] Kira K., Rendell L., Sleeman D., Edwards P. (1992). A practical appoach to feature selection. The Ninth International Workshop on Machine Learning.

[34] Williams G.J. (2009). Rattle: A data mining gui for R. R J..

[35] Breiman L. (2001). Random forests. Mach. Learn..

[36] Breiman L. (1996). Bagging predictors. Mach. Learn..

[37] McCulloch W.S., Pitts W. (1943). A logical calculus of the ideas immanent in nervous activity. Bull. Math. Biophys..

[38] Behrens T., Förster H., Scholten T., Steinrücken U., Spies E.D., Goldschmitt M. (2005). Digital soil mapping using artificial neural networks. J. Plant Nutri. Soil Sci..

[39] Vapnik V. (1995). The Nature of Statistical Learning Theory.

[40] Kampichler C., Wieland R., Calme S., Weissenberger H., Arriaga-Weiss S. (2010). Classification in conservation biology: A comparison of five machine-learning methods. Ecol. Inf..

[41] Foody G.M. (2004). Thematic map comparison: Evaluating the statistical significance of differences in classification accuracy. Photogramm. Eng. Remote Sensing.

[42] Viscarra Rossel R., Behrens T. (2010). Using data mining to model and interpret soil diffuse reflectance spectra. Geoderma.

[43] Van Groenigen J.W., Mutters C.S., Horwath W.R., van Kessel C. (2003). Nir and drift-mir spectrometry of soils for predicting soil and crop parameters in a flooded field. Plant Soil.

[44] Vinterbo S.A., Kim E.Y., Ohno-Machado L. (2005). Small, fuzzy and interpretable gene expression based classifiers. Bioinformatics.

[45] Choe E., van der Meer F., van Ruitenbeek F., van der Werff H., de Smeth B., Kim K.W. (2008). Mapping of heavy metal pollution in stream sediments using combined geochemistry, field spectroscopy, and hyperspectral remote sensing: A case study of the rodalquilar mining area, se spain. Remote Sensing Environ..

[46] Kemper T., Sommer S. (2004). Use fo airborne hyperspectral data to estimate residual heavy metal contamination and acidification potential in the guadiamar floodplain andalusia, spain after the aznacollar mining accident. Proc. SPIE.

[47] Stevens A., Udelhoven T., Denis A., Tychon B. (2010). Measuring soil organic carbon in croplands at regional scale using airborne imaging spectroscopy. Geoderma.

